# Evaluating Clinical Outcomes and Physiological Perspectives in Studies Investigating Respiratory Support for Babies Born at Term With or at Risk of Transient Tachypnea: A Narrative Review

**DOI:** 10.3389/fped.2022.878536

**Published:** 2022-06-23

**Authors:** Erin V. McGillick, Arjan B. te Pas, Thomas van den Akker, J. M. H. Keus, Marta Thio, Stuart B. Hooper

**Affiliations:** ^1^The Ritchie Centre, Hudson Institute of Medical Research, Melbourne, VIC, Australia; ^2^Department of Obstetrics and Gynaecology, Monash University, Melbourne, VIC, Australia; ^3^Division of Neonatology, Department of Pediatrics, Leiden University Medical Center, Leiden, Netherlands; ^4^Department of Obstetrics and Gynaecology, Leiden University Medical Center, Leiden, Netherlands; ^5^Athena Institute, Vrije Universiteit Amsterdam, Amsterdam, Netherlands; ^6^Newborn Research, The Royal Women’s Hospital, Melbourne, VIC, Australia; ^7^The Murdoch Children’s Research Institute, Melbourne, VIC, Australia; ^8^Department of Obstetrics and Gynaecology, University of Melbourne, Melbourne, VIC, Australia

**Keywords:** transient tachypnea of the newborn, respiratory distress, respiratory support, end-expiratory pressure, airway liquid

## Abstract

Respiratory distress in the first few hours of life is a growing disease burden in otherwise healthy babies born at term (>37 weeks gestation). Babies born by cesarean section without labor (i.e., elective cesarean section) are at greater risk of developing respiratory distress due to elevated airway liquid volumes at birth. These babies are commonly diagnosed with transient tachypnea of the newborn (TTN) and historically treatments have mostly focused on enhancing airway liquid clearance pharmacologically or restricting fluid intake with limited success. Alternatively, a number of clinical studies have investigated the potential benefits of respiratory support in newborns with or at risk of TTN, but there is considerable heterogeneity in study designs and outcome measures. A literature search identified eight clinical studies investigating use of respiratory support on outcomes related to TTN in babies born at term. Study demographics including gestational age, mode of birth, antenatal corticosteroid exposure, TTN diagnosis, timing of intervention (prophylactic/interventional), respiratory support (type/interface/device/pressure), and study outcomes were compared. This narrative review provides an overview of factors within and between studies assessing respiratory support for preventing and/or treating TTN. In addition, we discuss the physiological understanding of how respiratory support aids lung function in newborns with elevated airway liquid volumes at birth. However, many questions remain regarding the timing of onset, pressure delivered, device/interface used and duration, and weaning of support. Future studies are required to address these gaps in knowledge to provide evidenced based recommendations for management of newborns with or at risk of TTN.

## Introduction

Respiratory distress is most commonly associated with preterm newborns, due to lung immaturity, poor respiratory effort, and surfactant deficiency (1, 2). However, a growing number of otherwise healthy babies born at term (>37 weeks’ gestation) are presenting with respiratory distress symptoms within the first few hours of life, requiring admission to intensive care for treatment (3). These babies are commonly diagnosed with transient tachypnea of the newborn (TTN), which is characterized by tachypnea, expiratory braking, grunting and poor oxygenation (4, 5). TTN is often diagnosed retrospectively and historically has been considered a benign and self-limiting condition in most newborns. However, some can develop more severe respiratory distress and suffer serious morbidity including persistent pulmonary hypertension of the newborn (5).

**FIGURE 1:**
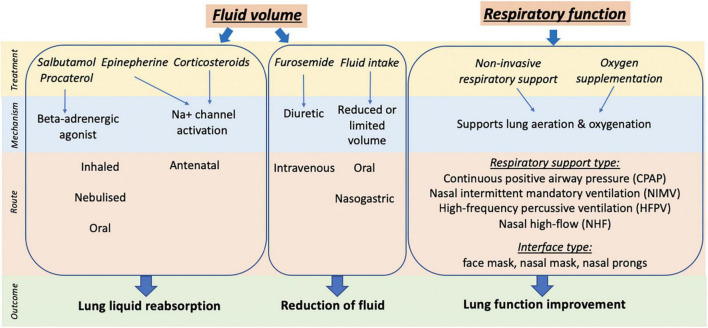
Summary of clinical treatment, mechanism, route of action and outcome targeted for management of TTN.

The incidence of TTN is greatest in babies born at term by cesarean section (CS) without labor and is increasing in parallel with the growing rates of elective CS worldwide (3, 6, 7). Historically, treatment of TTN has largely focused on targeting molecular airway liquid clearance mechanisms, which includes the pharmacological stimulation of sodium reabsorption with beta-receptor agonists and restricting newborn fluid intake (Figure 1). As determined by systematic review and meta-analysis, these methods have produced low-quality evidence over the years with limited impact on widespread management of the condition (8). These findings can be explained by recent evidence indicating that elevated airway liquid volumes, rather than delayed airway liquid clearance in the first few hours of life, cause the respiratory symptoms that characterize TTN after birth (9, 10). More liquid simply means that higher volumes of liquid need to be accommodated in lung tissue after birth (9, 10). This explains why newborns at greatest risk of TTN are those born at term by CS without labor. Newborns delivered by this method do not lose airway liquid via their nose and mouth during uterine contractions associated with labor, which is the only clearance mechanism that avoids liquid from entering lung tissue during lung aeration.

Recent studies have documented the pathophysiology associated with elevated airway liquid volumes at birth. These include:

1. The chest wall expands further and the diaphragm flattens (9), which reduces the newborn’s ability to inspire and increases the work of breathing.
2. Reduced end-expiratory lung volumes between breaths (functional residual capacity, FRC) (9, 11). As the reduction in FRC can be localized to lung regions with elevated liquid, this clearly demonstrates that the local clearance of liquid into lung tissue is the primary cause for the reduction in FRC (12).
3. Large reduction in lung compliance (9), which contributes to the reduced FRC and also limits inspiration.
4. Reduced respiratory gas exchange capacity, which increases the need for supplemental oxygen to maintain adequate oxygenation (13).
5. Reduced pulmonary blood flow (13) and increased pulmonary vascular resistance, potentially leading to persistent pulmonary hypertension of the newborn.
6. Induces tachypnea, grunting and expiratory braking in spontaneously breathing newborns, which are common physical symptoms observed in newborns with TTN (9, 11).

In recent years, clinical studies investigating the management of TTN have focused on providing respiratory support and the role of oxygen supplementation to support lung function (see Figure 1). However, while non-invasive respiratory support, particularly continuous positive airway pressure (CPAP), is currently the mainstay for managing TTN clinically, many questions remain and there is considerable heterogeneity in the approaches used. Importantly, use of respiratory support in term newborns with respiratory distress has been extrapolated from knowledge regarding respiratory support for preterm newborns. However, lung maturity and the pathophysiology underlying the respiratory distress are very different between preterm and term newborns. This narrative review synthesizes the current clinical evidence on the use of non-invasive respiratory support for term babies with or at risk of TTN. We have compared and contrasted study design, birth factors (gestational age, mode of delivery, and exposure to antenatal corticosteroids), application of respiratory support (timing, type, interface, device, and pressure) and clinical outcomes between studies. In addition, we provide insights into the current understanding of physiological mechanisms and impact of respiratory support in term newborns with TTN.

## Synthesis of Current Evidence

### Search Strategy

A literature search identified studies investigating the use of respiratory support in term newborns with or at high risk of TTN. Clinical studies published in English prior to January 2022 were evaluated and review articles were also examined to identify any relevant articles not captured by the electronic search.

A diagnosis of TTN represents a specific type of morbidity along the spectrum of respiratory distress severity. However, within a population of newborns with TTN there is significant heterogeneity in onset, severity and duration of symptoms. Thus, limiting this review to studies using respiratory support in the context of TTN, rather than any diagnosis of respiratory distress, enabled the heterogeneity in study characteristics and outcomes to be directly compared and contrasted. This allows a better understanding of the reported findings from studies investigating the respiratory management of newborns with or at risk of TTN.

### Tabulation of Study Factors

Study demographics (author, year, country, study type, and sample size), birth factors (gestational age, CS rate, and exposure to antenatal corticosteroids), characteristics for TTN diagnosis (physical characteristics, timing of tachypnea, imaging, and other parameters), timing of intervention (prophylactic or interventional), demographics of control and intervention groups (type of support, interface/device, and pressure), study aim, primary outcome, and overall study findings between groups were tabulated (Table 1). Studies are listed in chronological order to provide insights into approaches over time (Table 1).

**TABLE 1:** Clinical studies investigating the use of non-invasive respiratory support prophylactically in newborns at risk of TTN or interventional use in newborns diagnosed with TTN.

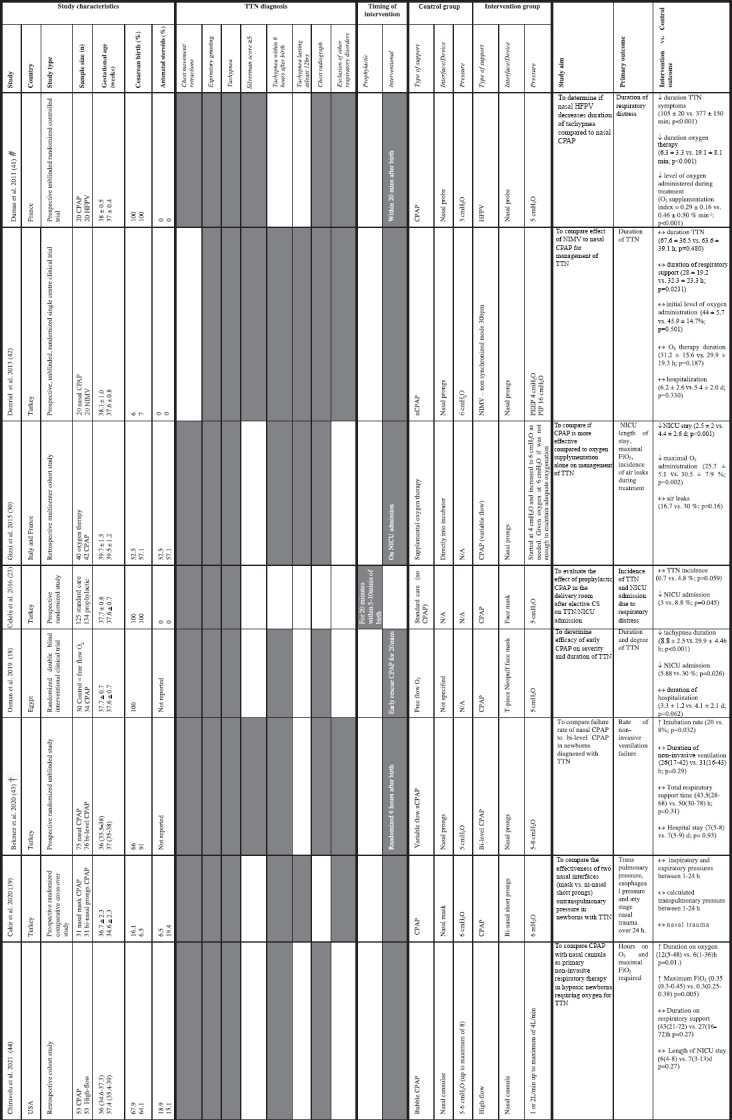

*Summary of study demographics, TTN diagnostic characteristics, variables in delivery of intervention and study outcome between standard management and intervention. Studies are listed in chronological order to provide insights into approaches over time. Characteristics reported as per individual studies are presented in table as text or grey shaded squares. Data were expressed as mean ± SD unless otherwise indicated as ^#^mean ± SEM; ^†^median (interquartile range). CPAP, continuous positive airway pressure; HFPV, high-frequency percussive ventilation; NIMV, nasal intermittent mandatory ventilation.*

### Study Demographics

Many studies have investigated treatments targeting sodium reabsorption and liquid volume on TTN management (Figure 1) (8, 14–17), with only eight studies investigating the use of non-invasive respiratory support (see Table 1), which were published between 2011 and 2021. The average gestational age at enrollment was early-term newborns (>37–38 + 6 weeks’ gestation), however, some studies did report newborns enrolled in groups which included some late preterm newborns (overall range 35–42 weeks’ gestation; see Table 1). The studies were conducted in hospitals in Egypt, France (two studies), Italy, Turkey (four studies) and the United States. Study types included six prospective studies (all were randomized) and two retrospective cohort studies (i.e., study design is not randomized). One was a multicenter cohort study while the remainder were single-center clinical studies. Studies compared outcomes between two different modes of respiratory support [continuous positive airway pressure (CPAP), nasal intermittent mandatory ventilation (NIMV), high frequency percussive ventilation (HFPV)], high-flow or supplemental oxygen exposure alone. One study was a comparative crossover investigating the influence of CPAP delivering device in the same newborns.

The studies varied in sample sizes from 40 to 259 newborns (average was 50). Three studies included babies born exclusively by CS, who are at greatest risk of developing TTN (gestational age range of newborns ∼36.5–38.5 weeks; see Table 1). The remaining studies investigated outcomes from babies with TTN born vaginally or by CS (CS rate ranged from 6 to 91% of those enrolled in individual studies; gestational age range of newborns ∼34.6–41 weeks). Antenatal corticosteroids were not used in two studies (gestational age range of newborns 36.5–39 weeks), whereas exposure ranged from 6.5 to 57.1% of newborns in other studies (gestational age range of enrolled newborns ∼32–42 weeks). Exposure to antenatal corticosteroids was not reported in two studies (gestational age range of newborns ∼35.5–39 weeks).

All studies reported outcomes in newborns displaying physical TTN symptoms and three used the Silverman or modified Silverman score (most commonly ≥5), which assesses respiratory distress severity in newborns without respiratory support. Six studies used tachypnea onset within 6 h and three studies used tachypnea lasting for at least 12 h to classify TTN. Six studies also relied on chest radiograph to confirm TTN, with varying numbers of pathological observations required for diagnosis.

### Non-invasive Respiratory Support Intervention Characteristics

Non-invasive respiratory support was given either prophylactically at birth (one study) or as an intervention following the development TTN after birth (seven studies). The largest study compared standard care (no respiratory support; *n* = 125) with prophylactic CPAP for 20 min initiated within 5-10 min of birth (*n* = 134) (18). The remaining studies provided non-invasive respiratory support to newborns with TTN at 20 min after birth (one study), on NICU admission (five studies; onset time unknown) or were randomized at 6 h after birth (one study). Two studies compared supplemental oxygen therapy with CPAP and five studies compared two different types of respiratory support for TTN (type/device/pressure). One study was a comparative crossover in newborns receiving respiratory support with two different devices (nasal mask vs. bi-nasal short prongs). Two studies provided CPAP for 20 min, using it as both a prophylactic and “early rescue” interventional therapy. In the remaining studies, the period of respiratory support was not set, but determined by the presence or absence of symptoms and ranged from 3 to 50 h (see Table 1).

### Method of Delivering Non-invasive Respiratory Support

Non-invasive respiratory support was provided by CPAP (eight studies), HFPV (one study), NIMV (one study), or high-flow (one study). CPAP was provided via nasal probe (one study), nasal prongs (five studies), face mask (three studies), or nasal mask (one study). In addition to standard CPAP (three studies), the type of CPAP used included bubble (one study), variable flow (two studies), and bi-level CPAP (one study). In the study that compared supplemental oxygen therapy with CPAP, oxygen was directly administered into an incubator equipped with an oxygenation sensor.

The CPAP level ranged between 5 and 8 cmH_2_O, while 4 cmH_2_O PEEP was administered during NIMV and 5 cmH_2_O administered during NHFPV. In one study, variable flow CPAP commenced with 4 cmH_2_O which was increased to a maximum of 6 cmH_2_O as needed and supplemental oxygen was provided if further assistance was required. One study investigating nasal high-flow provided 1 or 2 L/min up to a maximum of 4 L/min if required to support respiratory function. One study directly compared outcomes in the same newborns receiving 6 cmH_2_O CPAP, delivered either by nasal mask or bi-nasal short prongs.

### Outcomes of Studies Investigating the Effect of Respiratory Support in Babies With or at Risk of Transient Tachypnea of the Newborn

The primary outcome used in studies included the incidence of TTN (one study), the duration of TTN/respiratory distress (three studies), NICU administration with any respiratory symptoms (two studies), length of stay in NICU (one study), hours on oxygen (one study), maximal oxygen exposure (two studies), rate of failure of non-invasive respiratory support (one study), and incidence of air leaks (one study). The cross over study randomized newborns at a 6-h time point and investigated transpulmonary pressure, esophageal pressure and nasal trauma over 24 h.

The study which investigated prophylactic CPAP reported a lower incidence of TTN and an overall reduction in NICU admission in the CPAP group, however due to huge variability in the data only the NICU admission reached statistical significance. In the study that compared “early rescue” CPAP with free flow oxygen, CPAP decreased tachypnea duration and NICU admission, but not overall hospital stay. Four studies have compared two different modes of respiratory support. In newborns with TTN, nasal HFPV decreased duration of tachypnea and oxygen therapy compared to nasal CPAP when both were delivered at a pressure of 5 cmH_2_O. However, there was no difference in outcome of TTN duration or hospitalization between NIMV (PEEP 4 cmH_2_O) compared to CPAP (6 cmH_2_O) when delivered by nasal prongs. In a retrospective cohort study, newborns who received bubble CPAP (5–6 cmH_2_O; up to maximum of 8 cmH_2_O) via nasal prongs had a greater requirement for duration and total amount of oxygen despite no difference in length of NICU stay compared to newborns who received high flow (1–2 L/min up to maximum of 4 L/min). When variable flow CPAP using nasal prongs (5 cmH_2_O) was compared to bi-level CPAP (5–8 cmH_2_O) the need to intubate newborns was greater in the bi-level CPAP group, even though the duration of respiratory support and hospital stay were not different. Interventional use of CPAP (start at 4 cmH_2_O and increase to 6 cmH_2_O if required) was more effective than supplemental oxygen in newborns with TTN, reducing NICU stay and overall oxygen need. The crossover study reported that two nasal interfaces (mask vs. bi-nasal short prongs) had similar effects on transpulmonary pressure in newborns with TTN (19).

## Discussion

Our review has provided insights into the clinical evidence for using non-invasive respiratory support to manage term newborns with or at risk of TTN (Table 1). Our analysis builds upon a previous systematic review (20) which included three studies reporting outcomes using non-invasive respiratory support for TTN. They concluded that the evidence was limited and low-quality due to heterogeneity in study design and an inability to pool data. While this heterogeneity in study design remains a problem, we have included more recent publications to further explore the potential benefit of non-invasive respiratory support. While the outcomes appear to favor the use of non-invasive respiratory support for the treatment of TTN, the significant heterogeneity between studies currently is a limitation that makes direct comparisons between them difficult and precludes a rigorous assessment of the respiratory support approaches that can be used to treat or mitigate the risk of TTN. Furthermore, it is important to note that no studies were conducted in low- or middle-income countries and, as a result, these perspectives do not include an understanding of how TTN is or could be managed in these countries.

### Diagnosis and Severity of Transient Tachypnea of the Newborn

While all studies reported inclusion and exclusion criteria for TTN diagnosis, some relied on symptoms alone while others required further radiographic evidence or a scoring system (Silverman score) for diagnosis confirmation and severity assessment. One study reported the degree of respiratory distress as mild or severe, but did not stratify outcomes based on this clinical observation (18). It is possible that some selection bias may have occurred when enrolling newborns, as TTN is a diagnosis largely made retrospectively after the respiratory distress has resolved. As such, newborns with more severe or longer lasting respiratory distress symptoms are often excluded. These factors may explain the relatively short length of therapies and duration of hospital stay reported in the majority of studies, and color the perception that respiratory distress in term newborns is usually mild and self-resolving. However, these interventions remain a burden for families and health care systems. Indeed, this influence on diagnosis and inclusion criteria that may be partially based on outcomes could result in selection bias, particularly in retrospective cohort studies (2/8 studies included in this review; Table 1). Indeed, in these studies a TTN diagnosis will only be made if the newborn does not progress to more severe respiratory distress, whereas other newborns may have the same disease, but a more severe form that manifests into additional problems, including lung injury. This observation highlights the clinical problem faced when managing newborns born late preterm (>34 weeks gestation), early term (>37 weeks gestation), or full term (>39 weeks gestation) and who develop respiratory distress, as there is undoubtedly a spectrum of symptom severity. While the problem of diagnosis accuracy is not easy to counter, it is important to recognize this as a limitation. Future trials should take into consideration the reporting of factors such as severity of TTN based on symptoms (i.e., Silverman score), diagnostic tools (conventional X-ray or lung ultrasound) and degree of treatment provided to newborns. This will provide useful information for enabling comparison within and between studies. Large randomized trials which evaluate the spectrum of TTN severity and include outcomes in newborns that start with TTN but then develop more severe disease are also required to overcome the selection bias.

### Study Characteristics

#### Gestational Age

As most studies had small sample sizes (average 50 total newborns included), they had limited ability to stratify outcomes by subgroups, including that of gestational age, which could provide valuable insights into the respiratory support outcomes in subpopulations of newborns (21, 22). However, one study was large enough (259 newborns) to stratify into late preterm (34–36 + 6 weeks; 35 newborns) and early term (37–39 + 6 weeks; 224 newborns) comparing CPAP with standard care. They showed a significant difference in NICU admission across the entire cohort, which was driven largely by a difference in late preterm newborns (23). However, with regard to TTN incidence, while no significant difference between prophylactic CPAP and standard care was seen across the whole cohort (*p* = 0.059), a subgroup analysis revealed that the TTN incidence was greater in early-term (*p* = 0.057) compared late preterm newborns (*p* = 0.565) (23). Thus, future studies should avoid including newborns of a broad gestational age, without the capacity to stratify based on gestational age. Indeed, as the underlying cause and optimal treatments for the respiratory distress symptoms will likely differ in these two population sub-groups, mixing these subgroups will greatly reduce the effect size.

#### Mode of Birth

It is well established that delivery by CS in the absence of labor is a major risk factor for TTN (6), but despite this knowledge there was significant heterogeneity between studies for the inclusion of babies born by CS (range from 6 to 100%) and if the CS was planned or unplanned (usually in labor). Nevertheless, prophylactic CPAP (5 cmH_2_O) administration for 20 min within 5–10 min of birth in newborns born exclusively by CS (those at greatest risk of TTN) (23), was found to significantly reduce NICU admission, but had no statistically significant effect on TTN incidence (*p* = 0.059) (23). There was significant variability in the outcomes: the number needed to treat with non-invasive respiratory support to benefit newborns with TTN was 25 (OR 6.711; 95% CI: 0.795–55.55). Although the sample size in this study was one of the larger studies captured in this review (295 total newborns; 135 receiving CPAP), it is unclear how many of these newborns would be expected to have suffered from TTN. With a rate of ∼1 in 10 term babies born by CS expected to develop respiratory distress, the small but not significant difference in TTN or NICU admission is unsurprising. Nevertheless, it raises an important point about the efficacy of supporting newborns to clear airway liquid in the delivery room in order to mitigate the TTN symptoms that may develop and avoid the need to admit newborns into intensive care in the hours after birth. While the prophylactic CPAP study provides an important perspective into early intervention to reduce the risk of TTN, this study was undertaken in a teaching and research hospital in a high-income country. The logistical issues of applying prophylactic CPAP after CS birth in resource-limited settings and the large number of newborns needed to treat to show benefits, would likely preclude its use in less resourced hospitals and in low- and middle-income countries.

The physiological benefits of being exposed to labor and uterine contractions (i.e., CS with labor vs. elective CS) can reduce the risk of respiratory distress after a CS delivery, but still carries a greater risk compared to vaginal birth (6). As delivery by elective CS is the greatest risk factor for TTN, avoiding planned CS without medical indication would appear logical and is consistent with the World Health Organization recommendations (24). However, there is considerable heterogeneity between countries and hospitals as to the timing of planned CS deliveries, which can occur before 39 weeks, despite a higher risk of respiratory distress after birth. Furthermore, the increase in CS deliveries has led to an increase in the number of otherwise healthy babies, born at term, experiencing TTN. This is a growing concern, particularly as it is projected that by 2030, 28.5% of women worldwide will give birth by CS, equating to 38 million CSs annually, of which 33.5 million will be in low-middle income countries (7).

**FIGURE 2:**
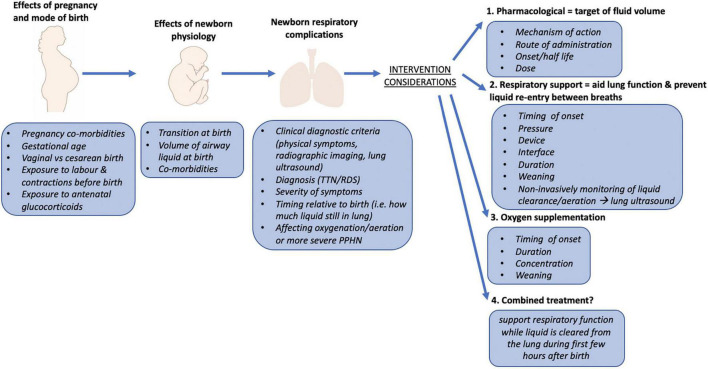
Overview of factors that influence the risk of respiratory distress in term newborns (>37 weeks gestation). Includes contributing factors during pregnancy, birth, and during the transition from fetal to newborn life. Consideration should be given to the physiology underpinning the newborn respiratory complications and the mechanisms underlying treatments that are provided to newborns to most successfully manage the respiratory distress symptoms. TTN, transient tachypnea of the newborn; RDS, respiratory distress syndrome; PPHN, persistent pulmonary hypertension of the newborn.

As it will always be necessary to deliver some newborns by CS, there is considerable merit in finding mechanisms to help prevent TTN from arising in the most at-risk newborns. As it is not feasible to adopt a wide-spread prophylactic approach (e.g., CPAP) for all CS births, a better understanding of the mechanisms causing respiratory distress in these newborns is required (Figure 2). In addition, identifying the specific risk conferred by CS birth, may allow identification of the sub-groups at greatest risk so that prophylactic treatment can be targeted to these newborns. Alternatively, rather than applying respiratory support to aid lung function before or after symptom onset, it may be possible to reduce the risk of TTN from developing by reducing airway liquid volumes at CS birth. This is the focus of a current clinical trial which aims to replicate the dorso-ventral spinal flexion usually achieved during uterine contractions following membrane rupture and amniotic fluid loss during CS delivery (Clinical Trial Number L74285.058.20). The resulting loss of liquid decreases the volume of liquid in the airways at birth, reduces the volume liquid that must absorbed into lung tissue and thereby reduces the risk of TTN. While further large studies are required, proposed approaches should also take into consideration applicability to both low-middle income settings as well as high-income countries.

#### Antenatal Corticosteroid Exposure

Some studies investigating respiratory morbidity after early term (37–39 weeks’ gestation) CS birth, specifically excluded newborns exposed to antenatal corticosteroids (22). In other studies, exposure to antenatal corticosteroids in newborns enrolled in individual studies ranged from 6 to 100%, which is consistent with the large variability in gestational age (32–42 weeks’ gestation) of enrolled newborns. While corticosteroids have been investigated for preventing respiratory morbidity in babies born by CS at term (25, 26), their use has been questioned (27) and systematically reviewed with a meta-analysis (28). Nevertheless, the underlying rationale for using antenatal corticosteroids and the precise mechanisms underlying its mode of action in TTN is unclear. It is assumed that they improve TTN outcome by accelerating lung maturity, enhancing liquid reabsorption, and reducing lung tissue density. However, there is some scientific evidence to indicate that reducing lung tissue density may increase the risk and severity of disease (11). It is possible that the large variability observed in both antenatal corticosteroid use and gestational age range of enrolled newborns within and between studies has compounded the heterogeneity in TTN incidence and outcomes between studies. Combining late preterm and term age groups into one cohort is problematic as it assumes that the underlying cause of the respiratory distress is similar and that the proposed treatment will be equally efficacious. Indeed, the evidence for administering corticosteroids steroids to women undergoing CS up to 39 weeks (28) is conflicting, with some obstetricians doubting the evidence at 37–39 weeks. Thus, we suggest that all future studies should report antenatal corticosteroid exposure in their cohort demographics and that the gestational age range be minimized so that findings can be more reliably interpreted.

### Respiratory Support Interventions for Babies With or at Risk of Transient Tachypnea of the Newborn

The majority of studies (7/8) captured in this review used respiratory support as an intervention in newborns diagnosed with TTN. However, the timing of onset markedly varied from 20 min after birth, at NICU admission or at 6 h after birth. As airway liquid clearance after birth increases lung interstitial tissue pressures (29), recent scientific evidence indicates that these elevated pressures play a key role in respiratory distress symptoms displayed by term or near-term newborns (9, 12). As a result, it is difficult to compare outcomes between studies when the lung is at different stages of transition, with respect to clearing this liquid from the airways and tissue (10). As such, reporting information about the timing of respiratory support onset and severity of disease is needed to better understand the underlying physiology of the newborns at the time of intervention and provide better context for the reported findings.

While all studies (Table 1) focused on newborns with or at risk of TTN, the large variation in study design makes it difficult to directly compare outcomes. Differences include (1) prophylactic CPAP vs. no intervention, (2) supplemental oxygen vs. CPAP, (3) different types and interfaces for applying the respiratory support, (4) different pressure levels, and (5) different timings for treatment onset and TTN diagnosis. Two studies (one randomized double blind clinical trial and one retrospective cohort study) indicate that CPAP may reduce tachypnea duration, NICU stay and maximal FiO_2_ exposure compared with oxygen alone (18, 30). However, it is difficult to draw conclusions with regard to the “early rescue” use of CPAP as the timing of application was not reported, particularly in regard to disease progression. As such, it remains unclear when CPAP should be applied. Importantly, these studies indicate that oxygenation in itself is not the main driver of TTN symptoms and that an end-expiratory pressure can mitigate disease progression. The findings are also consistent with the concept that CPAP helps to improve lung function when high volumes of liquid must be cleared over the first 4–6 h of life. While the initial oxygen levels provided and the amount of supplemental oxygen provided after CPAP levels were increased varied between studies, the FiO_2_ level was likely titrated against arterial oxygen saturations. Nevertheless, variability in these factors makes it difficult to tease apart the relative contribution of respiratory support and oxygenation for improving clinical outcomes in newborns with TTN.

Different respiratory support devices for TTN have been discussed previously (31) and different types of respiratory support have also been compared (five studies). All newborns in these studies received respiratory support by either nasal prongs or nasal mask (see Table 1), but variability in methods between studies precludes a rigorous comparison and not all studies were randomized. Unfortunately, no clinical studies have yet investigated the effect of different pressures with the same device to specifically examine the effect of pressure.

In the studies examined, the duration of respiratory support ranged from a few hours to a few days (3–50 h), which suggests that disease severity differed between studies, with some studies including those with more severe respiratory distress, as TTN is thought to resolve within the first day or so after birth. This heterogenous requirement for respiratory support may also reflect the intersection between gestational age dependent effects on lung immaturity and surfactant maturity and/or function on lung aeration. Thus, differences in disease severity, the timing of treatment onset relative to symptom onset and the rationale for providing treatment also reduces the capacity to compare outcomes between studies. However, as the findings indicate small but significant benefits of prophylactic or early CPAP use, larger studies with better defined inclusion criteria and treatment regimens are required. Indeed, while there was considerable heterogeneity in approaches, all studies reported that non-invasive respiratory support was well tolerated by newborns and was not associated with an increased risk of air leaks.

As for preterm newborns, it is important to identify the best methods for supporting term newborns with TTN and how the respiratory support provided facilitates or impedes the transitional changes in newborn physiology (Figure 2). This review has highlighted the need for future studies to identify interfaces, devices and pressures that most effectively support newborns with TTN, which may or may not be similar to what is used in premature newborns. However, while higher CPAP levels may reduce the need for supplemental oxygen (32), it is important to ensure that it does not impair cardiovascular function (2).

### Physiological Perspectives and Considerations for Treating the Underlying Cause of Transient Tachypnea of the Newborn

**FIGURE 3:**
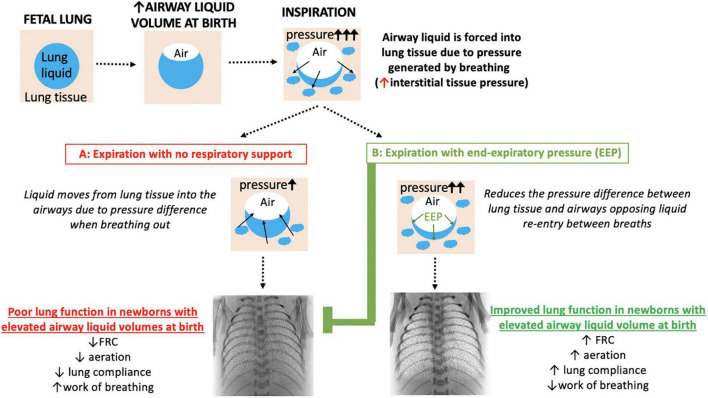
Schematic overview of how respiratory support with an end-expiratory pressure (EEP) supports lung function in near-term newborns with elevated airway liquid volumes at birth. Blue, lung liquid; beige, lung tissue. Included are synchrotron phase contrast X-ray images of newborn rabbits (equivalent to 38 weeks gestational age in terms of humans lung development) with elevated airway liquid volume at birth receiving respiratory support with no end-expiratory pressure (A: 0 cmH_2_O) or a positive end-expiratory pressure of 8 cmH_2_O (B) via mechanical ventilation to show the effect on lung aeration [white speckle pattern; reproduced with permission (12)]. FRC, functional residual capacity.

In contrast to premature newborns, little is known about the benefits of end-expiratory pressures to support lung function in term newborns with respiratory distress shortly after birth. Recent scientific studies indicate that greater airway liquid volumes at birth underpin the respiratory symptoms suffered by newborns with TTN, including tachypnea and expiratory grunting with rapid and labored breathing (9, 11). They have also shown that an end-expiratory pressure of 8 cmH_2_O (vs. 5 cmH_2_O) improves FRC and lung mechanics in mechanically ventilated newborn rabbits with airway liquid volumes similar to that expected following elective CS birth (∼37 ml/kg) (12). The higher end-expiratory pressures prevented liquid from re-flooding the airways between inflations, resulting in higher FRCs (Figure 3) and improved gas exchange (10) by increasing the lung’s surface area available for gas exchange throughout the respiratory cycle. However, the optimal end-expiratory pressures are still unknown and are likely to differ depending on disease severity and the volume of airway liquid present at birth.

Gestational age and exposure to antenatal corticosteroids are likely to be major complicating factors because the density of lung tissue relative to airspace volume is greatly reduced in a mature lung. As such, a larger volume of airway liquid must be accommodated by a relatively smaller volume of lung tissue, which likely explains the higher prevalence of TTN in mature term newborns. In contrast, when the lung is less mature a relatively smaller volume of airway liquid can be absorbed into a relatively greater tissue volume, which greatly reduces the impact on lung function (11). Thus, respiratory distress in preterm newborns (e.g., 32 weeks), which is sometimes classified as TTN because it is relatively mild, is likely to have a lung immaturity component to it. While it can also be treated with CPAP, the underlying cause is substantially different.

In addition to CPAP level, many other factors should be considered when assessing potential treatments for TTN. These include the timing of treatment onset after birth, the duration of treatment and the goals that can be used to indicate when support can be weaned (Figure 2). As a “one-size-fits-all” approach is unlikely to be sufficient (Figures 2, 3), there is a need to develop methods that provide greater clarity of treatment success and the ability to monitor progress over time. A recent study has investigated the use of non-invasive forced oscillometry to quantify respiratory mechanics in term newborns with TTN and may have some utility in assessing respiratory mechanics. However, as it was conducted in newborns on postnatal days 1 and 3, it is unclear how effective it is during the first day after birth (33). Furthermore, as lung ultrasound can monitor lung aeration in newborns (34) and assess the relative levels of airway liquid, it can identify pathological changes in lung function characteristic of TTN (35, 36). As lung ultrasound is non-invasive, does not involve radiation and can be conducted at the bedside with common ultrasound machines, it has much clinical utility. Lung ultrasound can detect TTN (37), monitor changes in lung function associated with repositioning newborns (38), monitoring clinical progression of TTN (39) and can potentially predict those newborns likely to require intensive care admission for TTN (40). As such, future studies aimed at investigating respiratory support approaches for newborns with TTN may benefit from utilizing lung ultrasound to more accurately assess the effectiveness of treatments.

## Conclusion

While the studies reviewed have shown that respiratory support interventions can improve outcomes for newborns with TTN, numerous questions remain unanswered. For instance, the timing of respiratory support onset (prophylactic/interventional), the method of support (device/interface), the pressure of support delivered and the approaches for weaning this support are currently unknown (Figure 2). When considering the best approach for supporting newborns with TTN, the mode of birth (elective CS vs. CS with labor vs. vaginal) and the time after birth of TTN symptom onset must also be considered as they will influence the success of treatment (Figure 2).

It is clear that further understanding of the continuum of respiratory distress severity and the mechanisms causing respiratory distress and TTN in term newborns is needed. While the most effective way to reduce the incidence of TTN is to avoid non-medically indicated elective CS deliveries, there is still a pressing need to find ways to best support newborns who develop TTN. Further large clinical trials are needed to identify approaches that reduce the risk of TTN in at-risk newborns or by better supporting respiratory function in newborns who develop TTN to minimize its severity.

Overall, this narrative literature review provides insights and considerations for future studies aimed at characterizing the effects of supporting term newborns with or at risk of TTN. This is an important step toward generating the highest quality evidence for guiding and informing recommendations for the most effective management strategies to improve outcomes for the growing burden of TTN in this group of otherwise healthy term babies.

## Author Contributions

EM, AP, and SH were responsible for the conception and design of the project and drafted the manuscript. EM conducted the data acquisition and synthesis. EM, AP, TA, JK, MT, and SH were involved in analysis and interpretation of the data. All authors contributed to the final version.

## Conflict of Interest

The authors declare that the research was conducted in the absence of any commercial or financial relationships that could be construed as a potential conflict of interest.

## Publisher’s Note

All claims expressed in this article are solely those of the authors and do not necessarily represent those of their affiliated organizations, or those of the publisher, the editors and the reviewers. Any product that may be evaluated in this article, or claim that may be made by its manufacturer, is not guaranteed or endorsed by the publisher.

## References

[1] Avery ME Mead J. Surface properties in relation to atelectasis and hyaline membrane disease. *AMA J Dis Child.* (1959) 97:517–23. 10.1001/archpedi.1959.02070010519001 13649082

[2] Martherus T Oberthuer A Dekker J Hooper SB McGillick EV Kribs A Supporting breathing of preterm infants at birth: a narrative review. *Arch Dis Child Fetal Neonatal Ed.* (2019) 104:F102–7. 10.1136/archdischild-2018-314898 30049727

[3] Clapp MA James KE Bates SV Kaimal AJ. Unexpected term NICU admissions: a marker of obstetrical care quality? *Am J Obstetr Gynecol.* (2019) 220:395. e1–12. 10.1016/j.ajog.2019.02.001 PMC8462396 30786256

[4] Chow SS Creighton P Kander V Haslam R Lui K. *2016 Report of the Australian and New Zealand Neonatal Network*. (2018). Sydney: ANZNN.

[5] Hansen AK Wisborg K Uldbjerg N Henriksen TB. Risk of respiratory morbidity in term infants delivered by elective caesarean section: cohort study. *BMJ.* (2008) 336:85–7. 10.1136/bmj.39405.539282.BE 18077440 PMC2190264

[6] Morrison JJ Rennie JM Milton PJ. Neonatal respiratory morbidity and mode of delivery at term: influence of timing of elective caesarean section. *BJOG.* (1995) 102:101–6. 10.1111/j.1471-0528.1995.tb09060.x 7756199

[7] Betran AP Ye J Moller A-B Souza JP Zhang J. Trends and projections of caesarean section rates: global and regional estimates. *BMJ Glob Health.* (2021) 6:e005671. 10.1136/bmjgh-2021-005671 34130991 PMC8208001

[8] Buchiboyina A Jasani B Deshmukh M Patole S. Strategies for managing transient tachypnoea of the newborn-a systematic review. *J Matern Fetal Neonatal Med.* (2016) 30:1524–32. 10.1080/14767058.2016.1193143 27762156

[9] McGillick EV Lee K Yamaoka S te Pas AB Crossley KJ Wallace MJ Elevated airway liquid volumes at birth: a potential cause of transient tachypnea of the newborn. *J Appl Physiol.* (2017) 123:1204–13. 10.1152/japplphysiol.00464.2017 28775070

[10] Hooper SB Te Pas AB Kitchen MJ. Respiratory transition in the newborn: a three-phase process. *Arch Dis Child Fetal Neonatal Ed.* (2016) 101:F266–71. 10.1136/archdischild-2013-305704 26542877

[11] te Pas AB Kitchen MJ Lee K Wallace MJ Fouras A Lewis RA Optimizing lung aeration at birth using a sustained inflation and positive pressure ventilation in preterm rabbits. *Pediat Res.* (2016) 80:85–91. 10.1038/pr.2016.59 26991259 PMC4973011

[12] McGillick EV te Pas AB Croughan MK Crossley KJ Wallace MJ Lee K Increased end-expiratory pressures improve lung function in near-term newborn rabbits with elevated airway liquid volume at birth. *J Appl Physiol.* (2021) 131:997–1008. 10.1152/japplphysiol.00918.2020 34351817

[13] Yamaoka S Crossley K McDougall A Rodgers K Zahra V Moxham A Increased airway liquid volumes at birth impairs cardiorespiratory function in preterm and near-term lambs. *J Appl Physiol.* (2022) 132:1080–90. 10.1152/japplphysiol.00640.2021 35271407

[14] Bruschettini M Moresco L Calevo MG Romantsik O. Postnatal corticosteroids for transient tachypnoea of the newborn. *Cochrane Database Syst Rev.* (2020) 2020:CD013222. 10.1002/14651858.CD013222.pub2 32180216 PMC7076329

[15] Moresco L Bruschettini M Macchi M Calevo MG. Salbutamol for transient tachypnea of the newborn. *Cochrane Database Syst Rev.* (2021) 2:CD011878. 10.1002/14651858.CD011878.pub3 PMC8094231 33543473

[16] Gupta N Bruschettini M Chawla D. Fluid restriction in the management of transient tachypnea of the newborn. *Cochrane Database Syst Rev.* (2021) 2:CD011466. 10.1002/14651858.CD011466.pub2 33599990 PMC8094737

[17] Vaisbourd Y Abu-Raya B Zangen S Arnon S Riskin A Shoris I Inhaled corticosteroids in transient tachypnea of the newborn: a randomized, placebo-controlled study. *Pediatr Pulmonol.* (2017) 52:1043–50. 10.1002/ppul.23756 28672098

[18] Osman AM El-Farrash RA Mohammed EH. Early rescue neopuff for infants with transient tachypnea of newborn: a randomized controlled trial. *J Matern Fetal Neonatal Med.* (2019) 32:597–603. 10.1080/14767058.2017.1387531 28965435

[19] Cakir U Yildiz D Okulu E Kahvecioglu D Alan S Erdeve O A comparative trial of the effectiveness of nasal interfaces used to deliver continuous positive airway pressure for a brief period in infants with transient tachypnea of the newborn. *Arch Bronconeumol.* (2020) 56:373–9. 10.1016/j.arbres.2019.07.027 31740083

[20] Moresco L Romantsik O Calevo MG Bruschettini M. Non-invasive respiratory support for the management of transient tachypnea of the newborn. *Cochrane Database Syst Rev.* (2020) 4:CD013231. 10.1002/14651858.CD013231.pub2 32302428 PMC7164572

[21] Bulut O Buyukkayhan D. Early term delivery is associated with increased neonatal respiratory morbidity. *Pediatr Int.* (2021) 63:60–4. 10.1111/ped.14437 32786118

[22] Thomas J Olukade TO Naz A Salama H Al-Qubaisi M Al Rifai H The neonatal respiratory morbidity associated with early term caesarean section–an emerging pandemic. *J Perinatal Med.* (2021) 49:767–72. 10.1515/jpm-2020-0402 33962503

[23] Celebi MY Alan S Kahvecioglu D Cakir U Yildiz D Erdeve O Impact of prophylactic continuous positive airway pressure on transient tachypnea of the newborn and neonatal intensive care admission in newborns delivered by elective cesarean section. *Am J Perinatol.* (2016) 33:099–106. 10.1055/s-0035-1560041 26295966

[24] Betran AP Temmerman M Kingdon C Mohiddin A Opiyo N Torloni MR Interventions to reduce unnecessary caesarean sections in healthy women and babies. *Lancet.* (2018) 392:1358–68. 10.1016/S0140-6736(18)31927-5 30322586

[25] Sotiriadis A Makrydimas G Papatheodorou S Ioannidis JP McGoldrick E. Corticosteroids for preventing neonatal respiratory morbidity after elective caesarean section at term. *Cochrane Database Syst Rev.* (2018) 8:CD006614. 10.1002/14651858.CD006614.pub3 PMC6513666 30075059

[26] Al Riyami N Al Hadhrami A Al Lawati T Pillai S Abdellatif M Jaju S. Respiratory distress syndrome in neonates delivered at term-gestation by elective cesarean section at tertiary care hospital in Oman. *Oman Med J.* (2020) 2020:e133. 10.5001/omj.2020.51 32607253 PMC7315520

[27] Arruda A Ormonde M Stokreef S Fraga B Franco C Dâmaso C Is there a role for antenatal corticosteroids in term infants before elective cesarean section? *Rev Bras Ginecol Obstet.* (2021) 43:283–90. 10.1055/s-0041-1726055 33979889 PMC10183905

[28] Saccone G Berghella V. Antenatal corticosteroids for maturity of term or near term fetuses: systematic review and meta-analysis of randomized controlled trials. *BMJ.* (2016) 355:i6416. 10.1136/bmj.i5044 27733360 PMC5062056

[29] Miserocchi G Poskurica BH Del Fabbro M. Pulmonary interstitial pressure in anesthetized paralyzed newborn rabbits. *J Appl Physiol.* (1994) 77:2260–8. 10.1152/jappl.1994.77.5.2260 7868443

[30] Gizzi C Klifa R Pattumelli MG Massenzi L Taveira M Shankar-Aguilera S Continuous positive airway pressure and the burden of care for transient tachypnea of the neonate: retrospective cohort study. *Am J Perinatol.* (2015) 32:939–43. 10.1055/s-0034-1543988 25811328

[31] Morley C. Which neonatal nasal CPAP device should we use in babies with transient tachypnea of the newborn? *J Pediatr.* (2011) 87:466–8. 10.2223/JPED.2146 22169882

[32] Martherus T Oberthuer A Dekker J Kirchgaessner C van Geloven N Hooper SB Comparison of two respiratory support strategies for stabilization of very preterm infants at birth: a matched-pair analysis. *Front Pediatr.* (2019) 7:3. 10.3389/fped.2019.00003 30761276 PMC6362425

[33] Klinger AP Travers CP Martin A Kuo H-C Alishlash AS Harris WT Non-Invasive forced oscillometry to quantify respiratory mechanics in term neonates. *Pediatr Res.* (2020) 88:293–9. 10.1038/s41390-020-0751-7 31935746 PMC7358118

[34] Blank DA Rogerson SR Kamlin COF Fox LM Lorenz L Kane SC Lung ultrasound during the initiation of breathing in healthy term and late preterm infants immediately after birth, a prospective, observational study. *Resuscitation.* (2017) 114:59–65. 10.1016/j.resuscitation.2017.02.017 28249708

[35] Ibrahim M Omran A AbdAllah N El-Sharkawy S. Lung ultrasound in early diagnosis of neonatal transient tachypnea and its differentiation from other causes of neonatal respiratory distress. *J Neonatal Perinatal Med.* (2018) 11:281–7. 10.3233/NPM-181796 30040751

[36] Liu J Chen X-X Li X-W Chen S-W Wang Y Fu W. Lung ultrasonography to diagnose transient tachypnea of the newborn. *Chest.* (2016) 149:1269–75. 10.1016/j.chest.2015.12.024 26836942

[37] Ma H-R Liu J Yan W-K. Accuracy and reliability of lung ultrasound to diagnose transient tachypnoea of the newborn: evidence from a meta-analysis and systematic review. *Am J Perinatol.* (2020). 10.1055/s-0040-1721134 33242910

[38] Louis D Belen K Farooqui M Idiong N Amer R Hussain A Prone versus supine position for lung ultrasound in neonates with respiratory distress. *Am J Perinatol.* (2019) 38:176–81. 10.1055/s-0039-1695776 31480084

[39] Li C-S Chu S-M Lien R Mok T-Y Hsu K-H Lai S-H. Prospective investigation of serial ultrasound for transient tachypnea of the newborn. *Pediatr Neonatol.* (2021) 62:64–9. 10.1016/j.pedneo.2020.09.002 32972849

[40] Poerio A Galletti S Baldazzi M Martini S Rollo A Spinedi S Lung ultrasound features predict admission to the neonatal intensive care unit in infants with transient neonatal tachypnoea or respiratory distress syndrome born by caesarean section. *Eur J Pediatr.* (2021) 180:869–76. 10.1007/s00431-020-03789-z 32949291 PMC7886822

[41] Dumas De La Roque E Bertrand C Tandonnet O Rebola M Roquand E Renesme L Nasal high frequency percussive ventilation versus nasal continuous positive airway pressure in transient tachypnea of the newborn: a pilot randomized controlled trial (NCT00556738). *Pediatr Pulmonol.* (2011) 46:218–23. 10.1002/ppul.21354 20963833

[42] Demirel G Uras N Celik IH Canpolat FE Dilmen U. Nasal intermittent mandatory ventilation versus nasal continuous positive airway pressure for transient tachypnea of newborn: a randomized, prospective study. *J Matern Fetal Neonatal Med.* (2013) 26:1099–102. 10.3109/14767058.2013.766707 23419098

[43] Bekmez BO Dizdar EA Büyüktiryaki M Sari F Uraş N Canpolat FE Comparison of nasal CPAP versus Bi-level CPAP in transient tachypnea of the newborn: a randomized trial. *Am J Perinatol.* (2020) 38:1483–7. 10.1055/s-0040-1713815 32594511

[44] Chiruvolu A Claunch KM Garcia AJ Petrey B Hammonds K Mallett LH. Effect of continuous positive airway pressure versus nasal cannula on late preterm and term infants with transient tachypnea of the newborn. *J Perinatol.* (2021) 41:1675–80. 10.1038/s41372-021-01068-9 33986469

